# Supplementary material for: Copper Phosphide Nanowires as High-Performance Catalysts for Urea-Assisted Hydrogen Evolution in Alkaline Medium
Source: Materials (Basel). 2023 Jun 3;16(11):4169. doi: 10.3390/ma16114169 (PMC10254407; doi:10.3390/ma16114169)
Supplement: Supplementary file 1 [file materials-16-04169-s001.zip › materials-2397942-supplementary.pdf]

Supporting Information

Copper Phosphide Nanowires as High-Performance Catalysts for Urea-Assisted Hydrogen Evolution in Alkaline Medium

Hui Shen [1], Tianran Wei [2], Junyang Ding [3,*] and Xijun Liu [2,*]

[1] School of Bioengineering, Hefei Technology College, Hefei 230012, China
[2] State Key Laboratory of Featured Metal Materials and Life-Cycle Safety for Composite Structures, School of Resources, Environment and Materials, Guangxi University, Nanning 530004, China
[3] Institute for New Energy Materials & Low-Carbon Technologies, School of Materials Science and Engineering, Tianjin University of Technology, Tianjin 300384, China
* Correspondence: junyangdingde18@163.com (J.D.); xjliu@tjut.edu.cn (X.L.)

Supplementary Figures

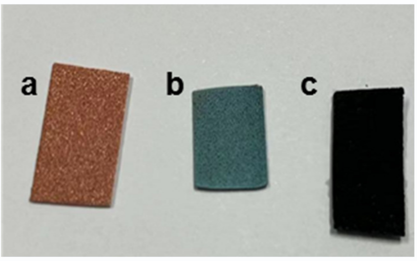

Figure S1. The optical pictures of copper foam (a), $Cu(OH)_2$-NW/CF (b), and Cu_3P-NW/CF (c).

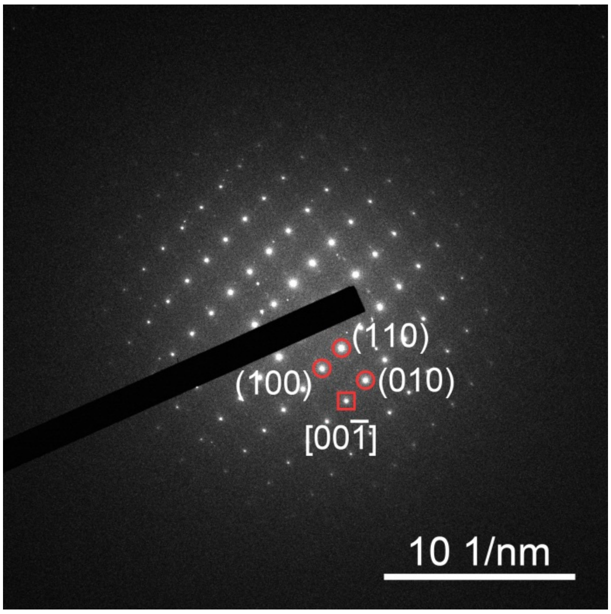

Figure S2. SAED pattern of Cu_3P-NW/CF.

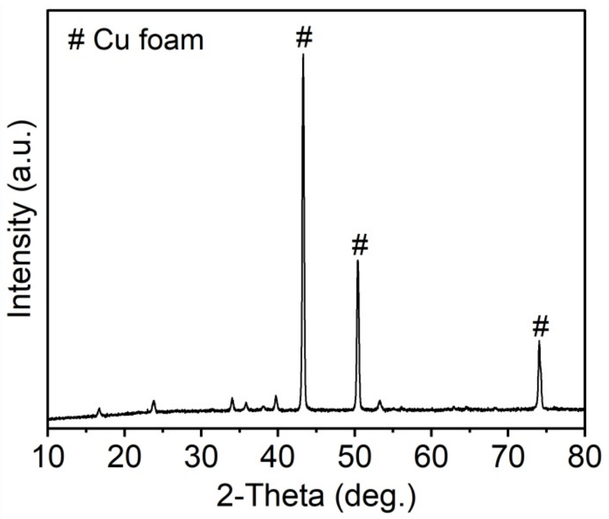

Figure S3. XRD pattern of $Cu(OH)_2$-NW/NF.

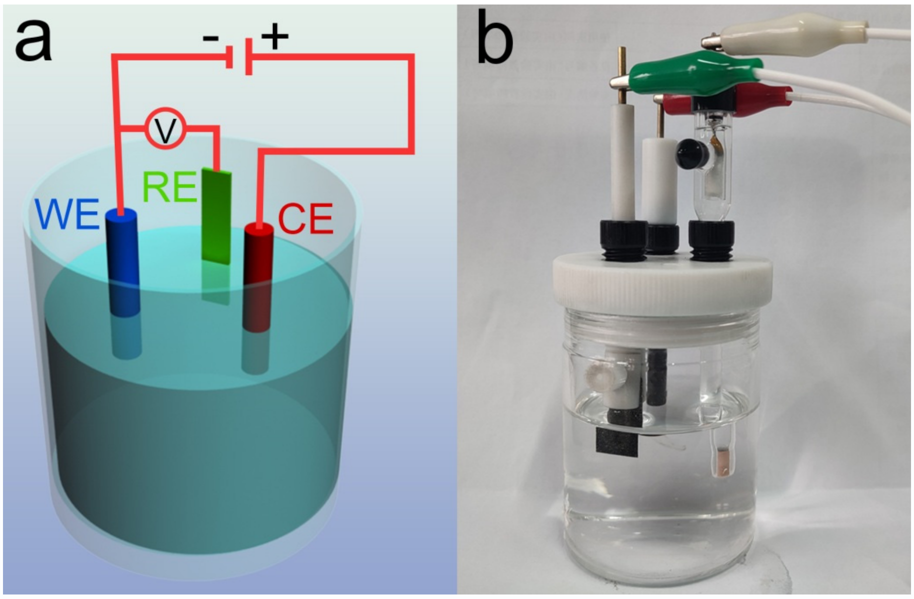

Figure S4. (a) Schematic diagram and (b) optical pictures of electrochemical tests.

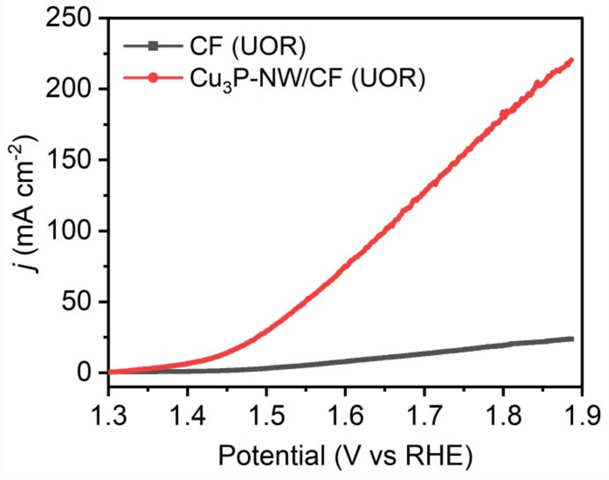

Figure S5. LSV curve of CF taken in 1.0 M KOH + 0.33 M urea.

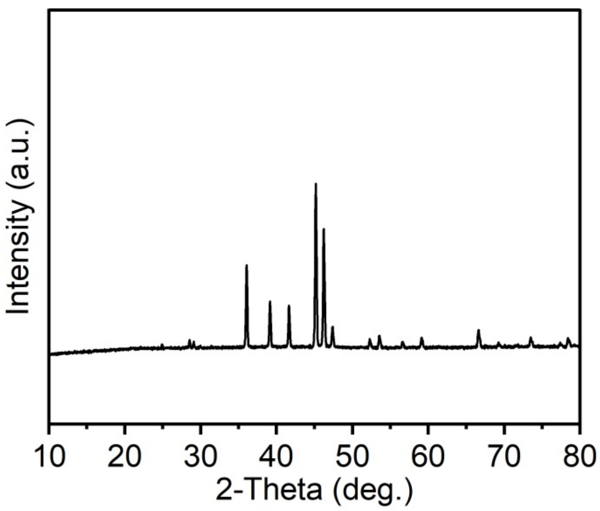

Figure S6. XRD pattern of Cu_3P-P/CF.

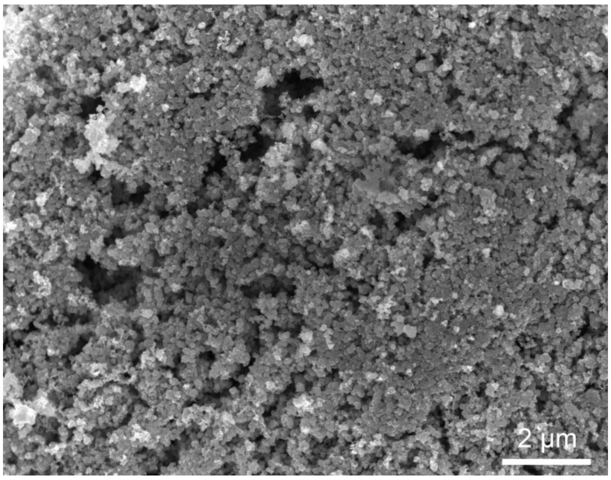

Figure S7. SEM iamge of Cu_3P-P/CF.

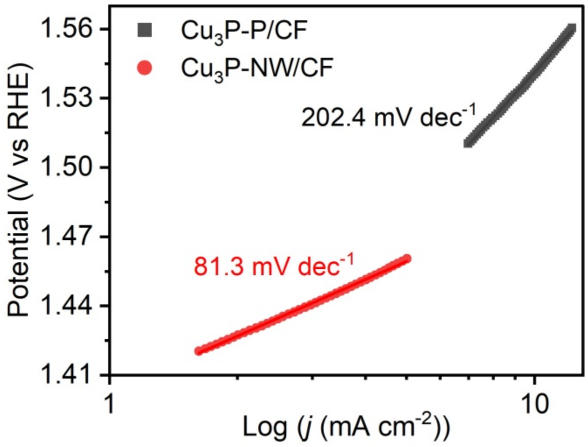

Figure S8. OER Tafel slopes of the two samples.

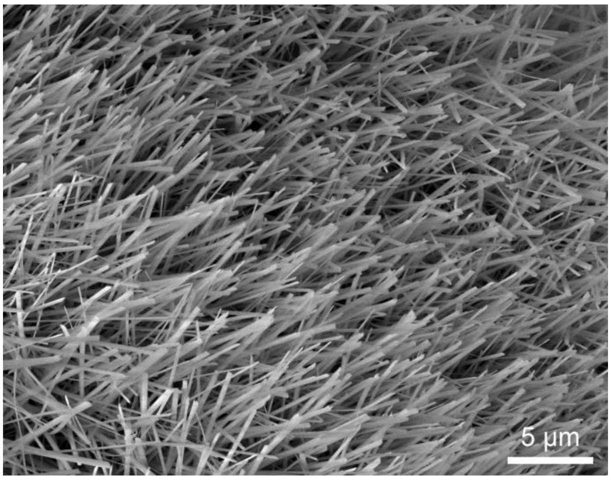

Figure S9. SEM image of Cu_3P-NW/CF recorded after the UOR stability test.

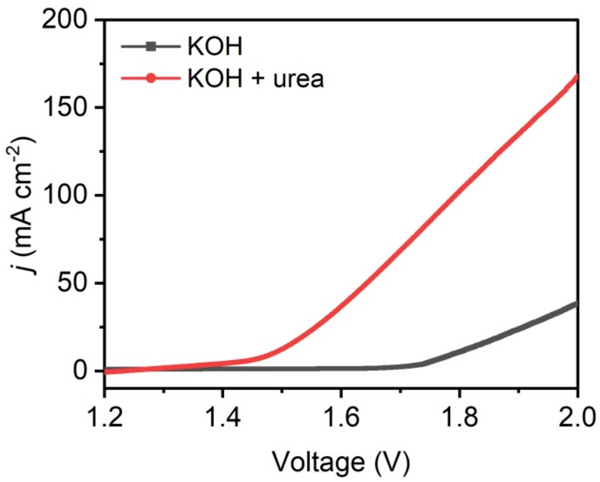

Figure S10. Polarization curves (without iR correction) of Cu_3P-NW/CF for two-electrode electrolyzer in different electrolytes.

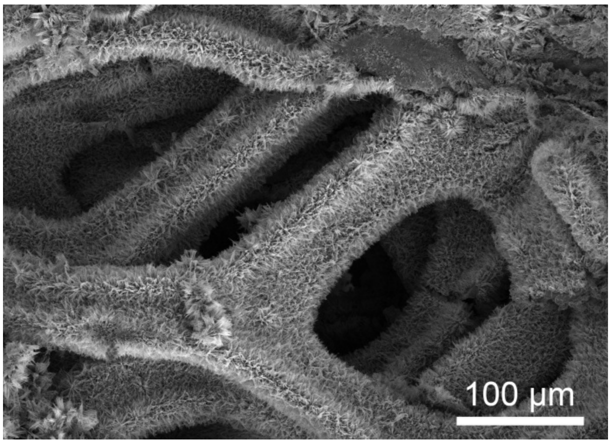

Figure S11. SEM image of Cu_3P-NW/CF for the two-electrode urea-assisted system after the 30 h stability test at 100 mA cm^{-2}.

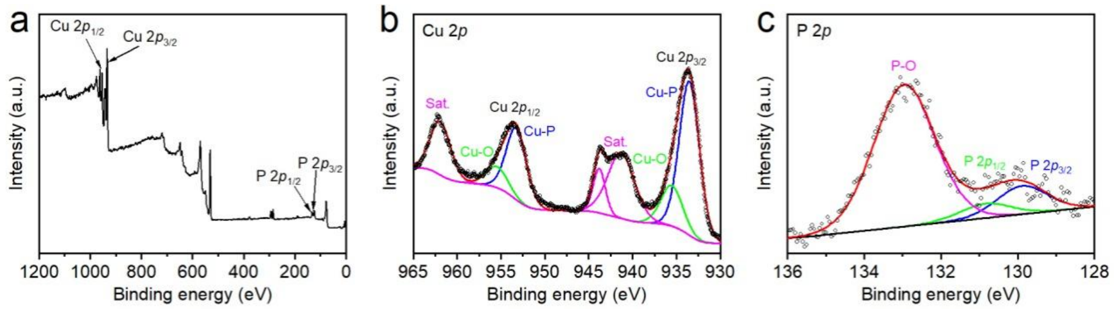

Figure S12. XPS analysis of Cu_3P-NW/CF for the two-electrode urea-assisted system after the 30 h stability test at 100 mA cm^{-2}. (a) XPS survey spectrum. High resolution (b) Cu 2*p* and (c) P 2*p* spectra.

Table S1. Comparison of the performance of urea-assisted electrolytic hydrogen production system with other reported catalysts.

Catalyst	Electrolyte	Cell voltage at 100 mA cm^{-2}	Stability	Ref.
Cu_3P-NW/CF	1 M KOH + 0.33 M urea	1.79 V	100 mA cm^{-2}, 30 h	This work
c-$CoNiP_x$/a-P-MnO_y	1 M KOH + 0.5 M urea	1.66 V	50 mA cm^{-2}, 40 h	Chem. Eng. J., 2023, 454, 140380
NC-FNCP	1 M KOH + 0.5 M urea	1.82 V	10 mA cm^{-2}, 20 h	Nano Res., 2022, 15, 1916
O-NiMoP	1 M KOH + 0.5 M urea	1.7 V	20 mA cm^{-2}, 10 h	Adv. Funct. Mater. 2021, 31, 2104951
U-NiMn-LDH/CFC	1 M KOH + 0.5 M urea	1.98 V	15 mA cm^{-2}, 40 h	Appl. Catal. A Gen., 2021, 614, 118049
$Co_{0.26}$-$Ni(OH)_2$ NPs/CF	1 M KOH + 0.5 M urea	1.79 V	10 mA cm^{-2}, 38 h	J. Catal., 2020, 381, 454
NiCoP/CC	1 M KOH + 0.5 M urea	1.8 V	20 mA cm^{-2}, 30 h	J. Mater. Chem. A 2019, 7, 9078
$Fe_{11.1\%}$–Ni_3S_2/NF	1 M KOH + 0.33 M urea	1.78 V	10 mA cm^{-2}, 20 h	J. Mater. Chem. A 2018, 6, 4346
